# 1-Methyl-2,6-*cis*-distyrylpiperidine

**DOI:** 10.1107/S1600536809049617

**Published:** 2009-12-09

**Authors:** Guangrong Zheng, Sean Parkin, Linda P. Dwoskin, Peter A. Crooks

**Affiliations:** a Department of Pharmaceutical Sciences, College of Pharmacy, University of Kentucky, Lexington, KY 40536, USA; b Department of Chemistry, University of Kentucky, Lexington, KY 40536, USA

## Abstract

The complete molecule of the title compound, C_22_H_25_N, is generated by crystallographic mirror symmetry, with two C atoms and the N atom lying on the mirror plane.  The central ring adopts a chair conformation and the dihedral angle between the aromatic rings is 56.69 (4)°.

## Related literature

The title compound is a *des*-oxygen lobeline derivative (Zheng *et al.*, 2005 ▶).
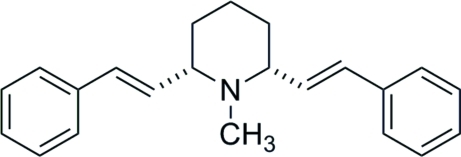


## Experimental

### 

#### Crystal data

C_22_H_25_N
*M* *_r_* = 303.43
Orthorhombic, 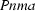

*a* = 17.3766 (2) Å
*b* = 18.1774 (6) Å
*c* = 5.5354 (7) Å
*V* = 1748.3 (2) Å^3^
*Z* = 4
Mo *K*α radiation
μ = 0.07 mm^−1^
*T* = 173 K
0.40 × 0.25 × 0.20 mm

#### Data collection

Nonius KappaCCD diffractometer
Absorption correction: none
12693 measured reflections
2077 independent reflections
1624 reflections with *I* > 2σ(*I*)
*R*_int_ = 0.044

#### Refinement

*R*[*F* ^2^ > 2σ(*F* ^2^)] = 0.051
*wR*(*F* ^2^) = 0.105
*S* = 1.10
2077 reflections
110 parameters
H-atom parameters constrained
Δρ_max_ = 0.19 e Å^−3^
Δρ_min_ = −0.16 e Å^−3^

### 

Data collection: *COLLECT* (Nonius, 1998 ▶); cell refinement: *SCALEPACK* (Otwinowski & Minor, 1997 ▶); data reduction: *DENZO-SMN* (Otwinowski & Minor, 1997 ▶); program(s) used to solve structure: *SHELXS97* (Sheldrick, 2008 ▶); program(s) used to refine structure: *SHELXL97* (Sheldrick, 2008 ▶); molecular graphics: *XP* in *SHELXTL* (Sheldrick, 2008 ▶); software used to prepare material for publication: *SHELXL97* and local procedures.

## Supplementary Material

Crystal structure: contains datablocks global, I. DOI: 10.1107/S1600536809049617/hg2598sup1.cif

Structure factors: contains datablocks I. DOI: 10.1107/S1600536809049617/hg2598Isup2.hkl

Additional supplementary materials:  crystallographic information; 3D view; checkCIF report

## Figures and Tables

**Fig. 1.**
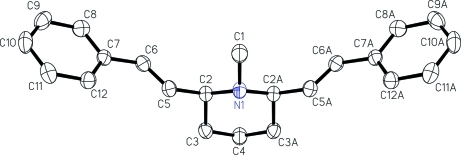
A view of the molecule with the atom numbering scheme (symmetry code A: x, -y+1/2,z). Displacement ellipsoids are drawn at the 50% probability level.

## supplementary crystallographic information

### Comment

1-Methyl-2,6-*cis*-distyrylpiperidine, C_22_H_25_N, is a *des*-oxygen lobeline derivative (Zheng *et al.*, 2005). The molecular structure is illustrated in Fig. 1, while selected geometric parameters are given in Table 1. The piperidine ring of the molecule is in the chair conformation and the *N*-methyl group is bonded equatorially to the piperidine ring. The styryl side chains are attached equatorially to the piperidine ring on atom C2 (and by symmetry, C2A). The molecule is mirror symmetric, with atoms N1, C1 and C4 lying on the mirror plane (Table 1). The double bond and the phenyl ring of the styryl side chain are approximately coplanar, as evidenced by the C5—C6—C7—C8 torsion angle [177.82 (12)°]. Moreover, the double bond is also approximately coplanar with C2—H2, as evidenced by the torsion angle C6—C5—C2—H2 [-4.66 (14)°].

### Experimental

The title compound was prepared from (-)-lobeline (Zheng *et al.*, 2005). Crystal suitable for X-ray diffraction studies were obtained by slow evaporation of an hexanes/ethylacetate (10:1) solution at room temperature.

### Refinement

H atoms were found in difference Fourier maps and subsequently placed in idealized positions with constrained distances of 0.98 Å (RCH_3_), 0.99 Å (*R*_2_CH_2_), 1.00 Å (*R*_3_CH), 0.95 Å (*R*_2_CH), 0.93 Å (N—H), and with *U*_iso_(H) values set to either 1.2*U*_eq_ or 1.5*U*_eq_ (RCH_3_) of the attached atom.

The SU of the torsion angle C6—C5—C2—H2 (-4.66 (14)°) is not directly available from the refinement because H2 is in a calculated position, as determined by a riding model. It was set equal to the SU of torsion angles C6—C5—C2—N1 and C6—C5—C2—C3, both of which were 0.14°. This is valid because the coordinates of H2 are determined geometrically from the coordinates of atoms C2, C5, N1 and C3.

### Crystal data

**Table:**

C_22_H_25_N	*F*(000) = 656
*M**_r_* = 303.43	*D*_x_ = 1.153 Mg m^−^^3^
Orthorhombic, *P**n**m**a*	Mo *K*α radiation, λ = 0.71073 Å
Hall symbol: -P 2ac 2n	Cell parameters from 12455 reflections
*a* = 17.3766 (2) Å	θ = 1.0–27.5°
*b* = 18.1774 (6) Å	µ = 0.07 mm^−^^1^
*c* = 5.5354 (7) Å	*T* = 173 K
*V* = 1748.3 (2) Å^3^	Irregular block, colourless
*Z* = 4	0.40 × 0.25 × 0.20 mm

### Data collection

**Table:**

Nonius KappaCCD diffractometer	1624 reflections with *I* > 2σ(*I*)
Radiation source: fine-focus sealed tube	*R*_int_ = 0.044
graphite	θ_max_ = 27.5°, θ_min_ = 2.2°
Detector resolution: 18 pixels mm^-1^	*h* = −22→22
ω scans at fixed χ = 55°	*k* = −23→23
12693 measured reflections	*l* = −7→7
2077 independent reflections	

### Refinement

**Table:**

Refinement on *F*^2^	Secondary atom site location: difference Fourier map
Least-squares matrix: full	Hydrogen site location: inferred from neighbouring sites
*R*[*F*^2^ > 2σ(*F*^2^)] = 0.051	H-atom parameters constrained
*wR*(*F*^2^) = 0.105	*w* = 1/[σ^2^(*F*_o_^2^) + (0.0317*P*)^2^ + 0.526*P*] where *P* = (*F*_o_^2^ + 2*F*_c_^2^)/3
*S* = 1.10	(Δ/σ)_max_ = 0.001
2077 reflections	Δρ_max_ = 0.19 e Å^−^^3^
110 parameters	Δρ_min_ = −0.16 e Å^−^^3^
0 restraints	Extinction correction: *SHELXL97* (Sheldrick, 2008), Fc^*^=kFc[1+0.001xFc^2^λ^3^/sin(2θ)]^-1/4^
Primary atom site location: structure-invariant direct methods	Extinction coefficient: 0.0098 (16)

### Special details

**Table:**

Geometry. All e.s.d.'s (except the e.s.d. in the dihedral angle between two l.s. planes) are estimated using the full covariance matrix. The cell e.s.d.'s are taken into account individually in the estimation of e.s.d.'s in distances, angles and torsion angles; correlations between e.s.d.'s in cell parameters are only used when they are defined by crystal symmetry. An approximate (isotropic) treatment of cell e.s.d.'s is used for estimating e.s.d.'s involving l.s. planes.
Refinement. Refinement of *F*^2^ against ALL reflections. The weighted *R*-factor *wR* and goodness of fit *S* are based on *F*^2^, conventional *R*-factors *R* are based on *F*, with *F* set to zero for negative *F*^2^. The threshold expression of *F*^2^ > σ(*F*^2^) is used only for calculating *R*-factors(gt) *etc*. and is not relevant to the choice of reflections for refinement. *R*-factors based on *F*^2^ are statistically about twice as large as those based on *F*, and *R*- factors based on ALL data will be even larger.

### Fractional atomic coordinates and isotropic or equivalent isotropic displacement parameters (Å^2^)

**Table:**

	*x*	*y*	*z*	*U*_iso_*/*U*_eq_	
N1	0.09202 (8)	0.2500	0.1454 (3)	0.0293 (4)	
C1	0.14851 (11)	0.2500	0.3409 (4)	0.0380 (5)	
H1A	0.1238	0.2500	0.5016	0.046*	
H1B	0.1809	0.2955	0.3259	0.046*	
C2	0.04374 (7)	0.18311 (6)	0.1549 (2)	0.0291 (3)	
H2	0.0129	0.1845	0.3073	0.035*	
C3	−0.01177 (8)	0.18156 (7)	−0.0582 (2)	0.0344 (3)	
H3A	0.0180	0.1770	−0.2100	0.041*	
H3B	−0.0453	0.1378	−0.0443	0.041*	
C4	−0.06143 (11)	0.2500	−0.0703 (4)	0.0397 (5)	
H4A	−0.0912	0.2500	−0.2228	0.048*	
H4B	−0.0983	0.2500	0.0659	0.048*	
C5	0.09154 (7)	0.11450 (7)	0.1566 (2)	0.0302 (3)	
H5	0.1269	0.1079	0.0274	0.036*	
C6	0.08859 (7)	0.06248 (7)	0.3230 (2)	0.0304 (3)	
H6	0.0547	0.0709	0.4547	0.036*	
C7	0.13201 (7)	−0.00733 (7)	0.3274 (2)	0.0280 (3)	
C8	0.12316 (7)	−0.05457 (7)	0.5236 (2)	0.0349 (3)	
H8	0.0885	−0.0419	0.6497	0.042*	
C9	0.16422 (8)	−0.11997 (7)	0.5374 (3)	0.0412 (4)	
H9	0.1574	−0.1517	0.6720	0.049*	
C10	0.21478 (8)	−0.13885 (7)	0.3562 (3)	0.0411 (4)	
H10	0.2430	−0.1835	0.3655	0.049*	
C11	0.22423 (8)	−0.09261 (7)	0.1615 (3)	0.0386 (4)	
H11	0.2591	−0.1055	0.0363	0.046*	
C12	0.18351 (7)	−0.02768 (7)	0.1467 (2)	0.0330 (3)	
H12	0.1907	0.0036	0.0111	0.040*	

### Atomic displacement parameters (Å^2^)

**Table:**

	*U*^11^	*U*^22^	*U*^33^	*U*^12^	*U*^13^	*U*^23^
N1	0.0269 (8)	0.0241 (8)	0.0369 (9)	0.000	−0.0038 (7)	0.000
C1	0.0347 (10)	0.0307 (10)	0.0486 (12)	0.000	−0.0101 (9)	0.000
C2	0.0291 (6)	0.0251 (7)	0.0332 (7)	−0.0001 (5)	0.0003 (5)	−0.0005 (5)
C3	0.0362 (7)	0.0278 (7)	0.0391 (8)	−0.0025 (6)	−0.0059 (6)	−0.0027 (6)
C4	0.0369 (11)	0.0326 (10)	0.0496 (12)	0.000	−0.0143 (9)	0.000
C5	0.0289 (6)	0.0273 (7)	0.0345 (7)	−0.0013 (5)	0.0019 (6)	−0.0016 (6)
C6	0.0279 (6)	0.0297 (7)	0.0334 (7)	−0.0020 (5)	0.0010 (5)	−0.0031 (6)
C7	0.0254 (6)	0.0242 (6)	0.0342 (7)	−0.0038 (5)	−0.0053 (5)	−0.0012 (5)
C8	0.0314 (7)	0.0371 (7)	0.0361 (8)	−0.0054 (6)	0.0003 (6)	0.0011 (6)
C9	0.0450 (8)	0.0315 (7)	0.0471 (9)	−0.0099 (6)	−0.0089 (7)	0.0125 (7)
C10	0.0385 (8)	0.0220 (7)	0.0628 (10)	0.0007 (6)	−0.0101 (7)	−0.0027 (7)
C11	0.0373 (8)	0.0303 (7)	0.0482 (9)	−0.0008 (6)	0.0012 (7)	−0.0079 (7)
C12	0.0362 (7)	0.0281 (7)	0.0348 (7)	−0.0031 (6)	−0.0005 (6)	0.0015 (6)

### Geometric parameters (Å, °)

**Table:**

N1—C1	1.461 (2)	C5—H5	0.9500
N1—C2^i^	1.4781 (14)	C6—C7	1.4765 (17)
N1—C2	1.4781 (14)	C6—H6	0.9500
C1—H1A	0.9877	C7—C12	1.3922 (17)
C1—H1B	1.0042	C7—C8	1.3930 (18)
C2—C5	1.4984 (16)	C8—C9	1.3885 (19)
C2—C3	1.5238 (17)	C8—H8	0.9500
C2—H2	1.0000	C9—C10	1.377 (2)
C3—C4	1.5155 (16)	C9—H9	0.9500
C3—H3A	0.9900	C10—C11	1.3765 (19)
C3—H3B	0.9900	C10—H10	0.9500
C4—C3^i^	1.5154 (16)	C11—C12	1.3785 (18)
C4—H4A	0.9900	C11—H11	0.9500
C4—H4B	0.9900	C12—H12	0.9500
C5—C6	1.3210 (17)		
			
C1—N1—C2^i^	110.79 (9)	C6—C5—C2	125.35 (12)
C1—N1—C2	110.79 (9)	C6—C5—H5	117.3
C2^i^—N1—C2	110.68 (13)	C2—C5—H5	117.3
N1—C1—H1A	112.1	C5—C6—C7	127.36 (12)
N1—C1—H1B	108.4	C5—C6—H6	116.3
H1A—C1—H1B	108.5	C7—C6—H6	116.3
N1—C2—C5	111.73 (10)	C12—C7—C8	117.85 (12)
N1—C2—C3	110.30 (11)	C12—C7—C6	123.03 (11)
C5—C2—C3	109.90 (10)	C8—C7—C6	119.10 (12)
N1—C2—H2	108.3	C9—C8—C7	120.93 (13)
C5—C2—H2	108.3	C9—C8—H8	119.5
C3—C2—H2	108.3	C7—C8—H8	119.5
C4—C3—C2	112.31 (11)	C10—C9—C8	120.09 (13)
C4—C3—H3A	109.1	C10—C9—H9	120.0
C2—C3—H3A	109.1	C8—C9—H9	120.0
C4—C3—H3B	109.1	C11—C10—C9	119.61 (13)
C2—C3—H3B	109.1	C11—C10—H10	120.2
H3A—C3—H3B	107.9	C9—C10—H10	120.2
C3^i^—C4—C3	110.34 (15)	C10—C11—C12	120.55 (13)
C3^i^—C4—H4A	109.6	C10—C11—H11	119.7
C3—C4—H4A	109.6	C12—C11—H11	119.7
C3^i^—C4—H4B	109.6	C11—C12—C7	120.97 (12)
C3—C4—H4B	109.6	C11—C12—H12	119.5
H4A—C4—H4B	108.1	C7—C12—H12	119.5
			
C1—N1—C2—C5	−54.16 (15)	C5—C6—C7—C12	−0.36 (19)
C2^i^—N1—C2—C5	−177.47 (8)	C5—C6—C7—C8	177.82 (12)
C1—N1—C2—C3	−176.71 (11)	C12—C7—C8—C9	−0.28 (18)
C2^i^—N1—C2—C3	59.97 (16)	C6—C7—C8—C9	−178.56 (11)
N1—C2—C3—C4	−56.00 (15)	C7—C8—C9—C10	0.3 (2)
C5—C2—C3—C4	−179.62 (12)	C8—C9—C10—C11	−0.1 (2)
C2—C3—C4—C3^i^	51.3 (2)	C9—C10—C11—C12	0.0 (2)
N1—C2—C5—C6	123.80 (14)	C10—C11—C12—C7	−0.02 (19)
C3—C2—C5—C6	−113.41 (14)	C8—C7—C12—C11	0.16 (18)
C2—C5—C6—C7	177.26 (11)	C6—C7—C12—C11	178.36 (11)

Symmetry codes: (i) *x*, −*y*+1/2, *z*.
